# Adaptive Fast-Slow Large Language Model Framework for Multidimensional Classification of Prenatal Ultrasound Reports: Comparative Study

**DOI:** 10.2196/91399

**Published:** 2026-05-28

**Authors:** Wei Zhong, Huihui Yan, Yifan Liu, Yan Liu, Kai Yang, Huimin Gao, Zhengyang Yao, Wenjing Hao, Yousheng Yan, Chenghong Yin

**Affiliations:** 1 Department of Medical Genetics, Beijing Obstetrics and Gynecology Hospital, Capital Medical University. Beijing Maternal and Child Health Care Hospital, Beijing, China; 2 Department of Prenatal Diagnosis Center, Beijing Obstetrics and Gynecology Hospital, Capital Medical University. Beijing Maternal and Child Health Care Hospital, Beijing, China; 3 Department of Central Laboratory, Beijing Obstetrics and Gynecology Hospital, Capital Medical University. Beijing Maternal and Child Health Care Hospital, No. 251 Yaojiayuan Road, Chaoyang District, Beijing, 100026, China, 86 15572779093

**Keywords:** large language models, prenatal ultrasound, DeepSeek, phenotype-driven diagnosis, chain-of-thought, retrieval-augmented generation

## Abstract

**Background:**

Phenotype-driven prenatal diagnosis relies on the precise correlation between ultrasound findings and genetic outcomes; however, this process is hindered by the unstructured nature of clinical ultrasound reports. While large language models (LLMs) hold the potential to address this challenge, their specific application in this domain remains systematically underexplored.

**Objective:**

To establish an effective LLM implementation framework for the clinical multidimensional classification of prenatal ultrasound reports, we evaluated the open-source DeepSeek-V3.2 family on real-world anomalous reports—covering both factual and subjective categories—while integrating retrieval-augmented generation (RAG) and chain-of-thought (CoT) reasoning.

**Methods:**

From a cohort of 4256 pregnancies, we extracted 254 reports with fetal anomalies. We comprehensively evaluated both the high-speed base model (DeepSeek-V3.2-B) and the reasoning-enhanced model (DeepSeek-V3.2-R) across all 5 classification dimensions, comprising 4 factual extraction tasks—primary classification, standardized terminology, anatomical system, and abnormality count—and 1 subjective severity assessment. We further explicitly evaluated the efficacy of RAG for the subjective tasks. Finally, to validate the clinical utility of this approach, we performed a correlation analysis between the expert-validated multidimensional phenotypic profiles and definitive genetic outcomes derived from amniocentesis.

**Results:**

While V3.2-B achieved high efficiency in factual tasks (accuracy and *F*_1_-score >90%), it underperformed in subjective severity grading (56.6% accuracy), exhibiting a recall of 0 for minor anomalies. Crucially, while RAG significantly improved both models’ performance on internal retrieval datasets (*P*<.05), this benefit did not generalize to external test datasets (*P*>.05). In contrast, the V3.2-R model utilizing CoT reasoning achieved superior robustness (86% accuracy and *F*_1_-score=0.75) on external data without RAG; notably, introducing RAG to V3.2-R degraded performance to 81%, suggesting potential noise interference. Clinical validation against amniocentesis outcomes confirmed that accurate multidimensional phenotypic profiles significantly stratified pathogenic genetic risks.

**Conclusions:**

The rapid base models are efficient for factual classification, and RAG enhances performance on data similar to the knowledge base, whereas CoT is indispensable for subjective assessment. Within the constraints of our dataset and current retrieval implementation, CoT proved more robust than RAG for subjective assessment. However, this finding is specifically tied to our experimental setup and should not be generalized as a universal conclusion. We recommend clinically adopting this adaptive “fast-slow” LLM framework to efficiently perform the multidimensional classification of prenatal ultrasound anomalies. This privacy-preserving, locally deployable solution provides a scalable path to accelerate phenotype-genotype research and optimize invasive diagnostic decision-making.

## Introduction

Prenatal ultrasound is fundamental for fetal assessment, but efficiently transforming these narrative reports into structured data for clinical decision-support remains a significant challenge [1,2]. While amniocentesis provides definitive genetic diagnoses, its associated risks of miscarriage and infection [3] make it unsuitable for universal application to all cases with abnormal ultrasound findings. Crucially, the decision to pursue invasive testing relies on a nuanced, multidimensional risk assessment rather than a singular finding. Different classification dimensions—specifically the affected anatomical system, the count of anomalies (isolated vs multiple), and the severity grading—each carry distinct predictive weights regarding chromosomal outcomes. For instance, multisystem defects or lethal malformations suggest a significantly higher genetic risk compared to isolated soft markers.

Therefore, a comprehensive integration of these multidimensional classifications is essential to provide patients with data-driven counseling, enabling them to weigh the probability of a genetic disorder against the procedural risks of amniocentesis. However, the large-scale analysis required to validate and refine this multidimensional risk stratification is hindered by the nature of clinical documentation: the frequent occurrence of benign anomalies and inconsistent descriptive terminology [3-7] creates unstructured “data silos.” Establishing a standardized framework to correlate these specific sonographic phenotypes with genetic outcomes is vital; yet, it has been hindered by the labor-intensive, expert-dependent process of annotating large-scale datasets.

The recent emergence of high-performance, open-source large language models (LLMs) offers a potential solution to this clinical natural language processing bottleneck [8-11]. However, the deployment of LLMs in medicine faces the critical challenge of hallucinations [12]. To mitigate this, retrieval-augmented generation (RAG) has become the prevailing paradigm, anchoring model outputs to external knowledge bases to ensure factual accuracy [13]. However, RAG primarily enhances information retrieval rather than logical reasoning. For complex, subjective tasks—such as assessing the severity of fetal anomalies based on subtle descriptive nuances—access to external knowledge may be insufficient without the capacity for deep reasoning. This has catalyzed the development of chain-of-thought (CoT) reasoning models [12,14], which mimic the deliberate “System 2” thinking popularized by Daniel Kahneman [15,16] by decomposing complex problems into intermediate logical steps. Unlike proprietary models, modern open-source LLMs, such as DeepSeek-V3.2, can be securely deployed within hospital environments. The V3.2 iteration uniquely offers both a high-speed base model (V3.2-B) and a reasoning-enhanced variant (V3.2-R), presenting a new opportunity to address the varying complexity of medical tasks—ranging from routine information extraction to complex logic-based severity assessment—within a unified local framework.

Despite these technological advances, the comparative effectiveness of retrieval-based versus reasoning-based approaches in automating the analysis of prenatal ultrasound reports remains largely unexplored. This study proposes an adaptive “fast-slow” LLM framework to address the multidimensional complexity of fetal phenotype extraction. We utilized the DeepSeek-V3.2 suite to evaluate the trade-offs between the fast base model (V3.2-B) and the slow reasoning model (V3.2-R), specifically assessing the utility of RAG versus CoT in subjective severity grading. To establish the clinical validity of this multidimensional classification, we further analyzed the association between expert-verified results and “gold standard” genetic outcomes from amniocentesis. Our objective was to demonstrate that accurate, multidimensional profiling is strongly predictive of pathogenic risks, and that while the fast base LLM and RAG suffice for factual tasks, CoT reasoning is indispensable for automating the subjective components of this profile, thereby accelerating phenotype-driven diagnosis.

## Methods

### Patient Recruitment and Data Collection

**Figure 1.**
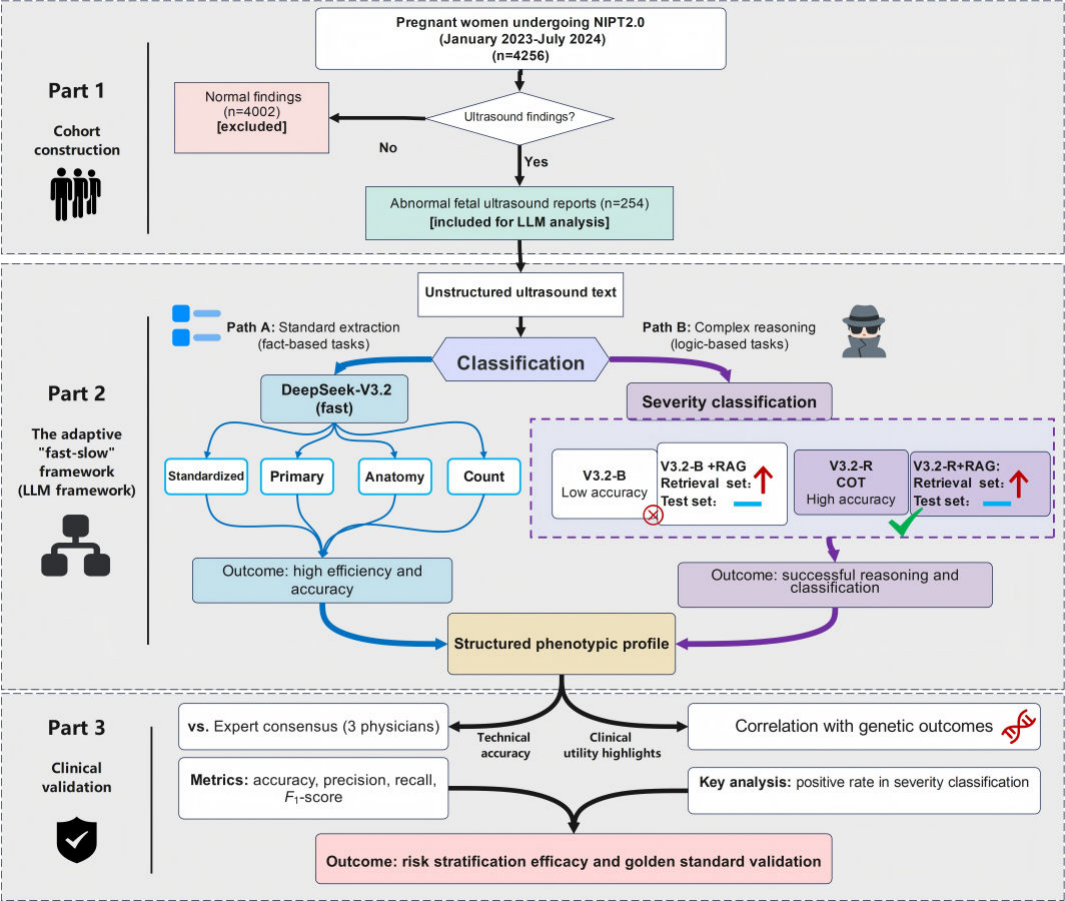
Flowchart of the study design. The workflow comprises three stages: (1) cohort construction: the selection of 254 abnormal fetal ultrasound reports from 4256 screenings. (2) The adaptive “fast-slow” framework: a dual-pathway system deploying DeepSeek-V3.2-B (fast) for objective factual extraction and DeepSeek-V3.2-R (slow) for subjective severity assessment. This phase explicitly compares the efficacy of RAG versus CoT reasoning. (3) Clinical validation: the dual assessment of the structured output against expert consensus (technical accuracy) and amniocentesis genetic outcomes (clinical utility). CoT: chain-of-thought; LLMs: large language models; NIPT2.0: noninvasive prenatal test; RAG: retrieval-augmented generation; V3.2-B: DeepSeek-V3.2 base model; V3.2-R: DeepSeek-V3.2 reasoning-enhanced model.

Between January 2023 and July 2024, 4256 pregnant women underwent an enhanced noninvasive prenatal test (NIPT2.0) as part of a longitudinal clinical efficacy validation study. The inclusion criteria were singleton pregnancies between 12 and 20 weeks of gestation, while excluding those with conditions known to significantly interfere with the NIPT2.0 analysis. This specific gestational window was deliberately selected because it aligns with the optimal and most actionable clinical timeframe for evaluating the necessity of invasive amniocentesis. Although this specific window inherently excludes late-onset anomalies presenting in the third trimester, it precisely captures the most critical period for phenotype-driven genetic risk assessment. Furthermore, because the NIPT2.0 screening—which covers both common aneuploidies and select monogenic disorders [17]—was offered free of charge, participation rates were exceptionally high. This provided a broad and highly representative real-world sample of early-to-mid second-trimester ultrasound findings, laying a solid data foundation for the downstream phenotype-genetic correlation analysis. Maternal age, family history, and prenatal ultrasound reports were collected at enrollment. Participants with negative (low-risk) NIPT2.0 results received routine follow-up, while those with positive (high-risk) results were advised to undergo amniocentesis for definitive diagnosis. The manual review of all records identified 254 participants with ultrasound reports showing anomalies of the fetus, placenta, umbilical cord, or amniotic fluid. These 254 reports constituted the dataset for this analysis. The study workflow is depicted in Figure 1.

### Multidimensional Classification of Prenatal Ultrasound Reports by LLMs

The 254 selected abnormal prenatal ultrasound reports were organized into a spreadsheet format. We utilized Dify (LangGenius, Inc) [18] to call the application programming interface of DeepSeek-V3.2, provided by the SiliconCloud service [19], to perform the automated classification. Each report was analyzed individually in a single-turn conversation with model parameters set to temperature=0 and top-p=0.95.

Five classification schemes were developed to structure the raw report data, including 4 fact-based classifications and 1 subjective assessment. For each scheme, prompts were designed by a sonographer and a prenatal diagnostician to guide the LLM. Due to their extensive length, the full verbatim prompts are provided in Multimedia Appendix 1. Nevertheless, all prompts adhered to a consistent design framework; they explicitly delineated the clinical rules and definitions for each classification scheme (as outlined below) and strategically incorporated specific explanations alongside clinical examples to disambiguate potentially confusing or overlapping findings. For classification purposes, suspected anomalies were treated as confirmed. The 5 schemes were as follows:

1. Primary classification: categorized reports into 4 mutually exclusive groups: increased nuchal translucency (NT), other soft markers, structural abnormalities, and fetal growth restriction (FGR).
2. Standardized terminology: converted descriptive text into standardized clinical terms while omitting laterality (eg, standardizing “left ventriculomegaly” to “ventriculomegaly”).
3. Anatomical system: mapped anomalies to the affected fetal system (eg, nervous and cardiovascular). Nonstructural or nonfetal findings were classified as “None.”
4. Abnormality count: Classified reports as “Solitary” (a single anomaly) or “multiple” (≥2 distinct anomalies, including bilateral presentations of a single finding).
5. Severity (subjective assessment): Findings were graded based on the most severe anomaly present, categorized into 3 levels according to standardized clinical criteria: lethal (eg, anencephaly, typically requiring termination of pregnancy), major (eg, complex congenital heart disease, significantly affecting viability), or minor (eg, cleft lip, surgically correctable postnatally). Nonstructural findings, such as NT or FGR, were categorized as “Other.”

For all schemes except “abnormality count” and “severity,” the model could generate multiple comma-separated outputs per report.

### LLM Execution and Evaluation

Two DeepSeek models were utilized sequentially. While both models belong to the DeepSeek-V3.2 family and share a highly efficient underlying architecture (incorporating mechanisms like DeepSeek Sparse Attention for rapid processing), their posttraining paradigms and inference mechanisms differ fundamentally [20]. V3.2-B is optimized using standard supervised fine-tuning for rapid instruction following and direct pattern matching, making it highly efficient for objective factual extraction. In contrast, V3.2-R is a reasoning-enhanced variant heavily trained with a scalable reinforcement learning protocol to intrinsically generate intermediate CoT steps. During inference, V3.2-R allocates substantial additional computational resources to process an internal “thinking” phase before producing the final response. This architectural distinction mimics human “System 2” deliberate deduction, enabling it to handle subjective clinical nuances, albeit at the cost of significantly longer processing times.

First, the V3.2-B rapidly processed all reports for initial classification (2‐4 s/report). To ensure the reliability of the classification, 2 experienced attending prenatal diagnosticians independently evaluated the V3.2-B outputs in a double-blind manner, labeling each classification as “Correct” or “Incorrect.” Disagreements were resolved through consensus meetings guided by a senior chief diagnostician (YY) to form a preliminary reference standard. Because all discrepancies were ultimately resolved via 100% consensus, formal interrater reliability metrics were not calculated.

Subsequently, the senior chief diagnostician utilized this preliminary standard to evaluate V3.2-R outputs. This secondary expert review step was essential because V3.2-R occasionally presented valid but differently phrased classifications that required expert judgment rather than simple string-matching, thereby establishing the final “gold standard” dataset.

Both models were independently evaluated on the entire dataset across all 5 classification schemes. For this study, an *F*_1_-score greater than 0.90 was prospectively defined as indicating highly reliable and clinically acceptable performance for the classification of prenatal ultrasound anomalies.

### Construction of Knowledge Base and Implementation of RAG

To evaluate the efficacy of RAG in enhancing model performance, the dataset of expert-verified severity assessments (n=254) was systematically partitioned based on sequential identification numbers. The first half (n=127) constituted the retrieval set, which was vectorized to construct the external knowledge base. The subsequent half (n=127) served as the unseen test set. While this sequential split shares local linguistic patterns and clinical workflows, it was intentionally designed to simulate a real-world scenario, in which a hospital utilizes its own historical records as a RAG knowledge base to process new, incoming reports of a similar style.

The RAG pipeline was deployed using the Dify platform. We integrated the Qwen3-Reranker-8B model for semantic reranking of candidate chunks. The retrieval parameters were configured with a Top-K of 3 and a similarity score threshold of 0.60 to filter out low-relevance noise. Finally, both V3.2-B and V3.2-R were tested on both the retrieval set and the test set to assess two critical metrics: (1) the effectiveness of RAG in retrieving and utilizing “seen” knowledge (performance on the retrieval set) and (2) the generalizability of the RAG-enhanced models when applied to “unseen” clinical data (performance on the test set).

### Follow-Up and Prenatal Diagnostic Outcomes

Among the 254 women with abnormal ultrasound findings, those with high-risk NIPT2.0 results were counseled for diagnostic amniocentesis. Definitive genetic testing included one or more of the following: karyotyping, chromosomal microarray analysis, whole-exome sequencing, and copy number variation sequencing. Cases were categorized based on these results. Patients who declined diagnostic testing after a high-risk NIPT2.0 result were excluded from the association analysis.

For the purpose of this study, a negative prenatal diagnostic outcome was assigned to all participants with a low-risk NIPT2.0 result. This approach was justified by the high negative predictive value of NIPT2.0 and supported by two observations in our cohort: (1) all women in this group who nonetheless underwent amniocentesis for sonographic indications had negative results and (2) clinicians did not recommend invasive testing for the remainder, judging the genetic risk to be low.

### Association Analysis Between Classified Ultrasound Abnormalities and Genetic Outcomes

An association analysis was performed on 251 eligible cases, correlating the classified ultrasound abnormalities with genetic diagnostic outcomes. This analysis utilized a “gold standard” dataset, which consisted of clinician-verified LLM classifications supplemented with manual corrections. The results were visualized using bar charts, showing the number and proportion of positive and negative genetic diagnoses for each anomaly category.

Prior to analysis, manual data curation was performed to standardize the LLM outputs for 3 classification schemes (standardized terminology, primary classification, and anatomical system). This step involved consolidating semantically identical but textually variant terms (eg, merging “Increased NT” and “Increased nuchal translucency”) and grouping identical combinations of findings listed in different orders to ensure accurate frequency counts for the association analysis.

### Statistical Analysis

To evaluate the performance of DeepSeek-V3.2, we calculated the overall accuracy for the categories. A true positive is recorded when an item in the model’s output list also appears in the gold standard; a false positive is an output item absent from the gold standard; a false negative is a gold-standard item missing from the output list. The *F*_1_-score was calculated as follows:


$$F_{1}=2\times \frac{Precision\times Recall}{Precision+Recall}$$

For the multiclass “Severity” classification, macroaveraged precision, recall, and *F*_1_-scores were calculated to assess balanced performance across all categories:


$$Macro-F_{1}=\frac{1}{N}\sum_{i=1}^{N}F_{1_{i}}$$

where *N* is the number of classes. Additionally, we calculated the precision, recall, and *F*_1_-score for each of the 4 severity categories individually.

For the association analysis, Pearson’s *χ*^2^ test was used to compare the rate of positive genetic diagnoses between the solitary and multiple abnormality categories, with significance set at *P*<.05. Due to the limited number of positive cases within the numerous categories of the other classifications, these were analyzed using descriptive statistics. The results were visualized as the number and proportion of cases with positive versus negative genetic diagnoses for each abnormality type.

The difference in accuracy between the pre- and post-RAG phases was analyzed using the McNemar test for paired categorical data. Continuity correction was applied where appropriate. Statistical significance was defined as a 2-sided *P*<.05. Data analysis was conducted using Python 3.10.

### Ethical Considerations

This comparative effectiveness study utilized the existing data from a prospectively registered cohort. The cohort protocol was registered with the Medical Research Registration and Filing Information System of the National Health Security Information Platform of China (registration number MR-11-24-002508). The study was conducted in accordance with the Declaration of Helsinki and approved by the institutional review board (2023-KY-099‐02). All participants in the original cohort provided written informed consent, agreeing to the use of their deidentified clinical information for scientific research purposes. As this study constitutes a secondary analysis of existing, deidentified data for comparative effectiveness purposes without causal inference, no additional patient contact or consent was required.

## Results

### Screening and Identification of Cases With Positive Amniocentesis Results

Of the 254 women with abnormal ultrasound findings, 30 had high-risk NIPT2.0 results. Three of these women, all with a high risk for Trisomy 21, declined amniocentesis and were therefore excluded from the association analysis. The remaining 27 women underwent amniocentesis, all of whom were confirmed to have a positive genetic diagnosis. This resulted in a final cohort of 251 cases for the association analysis, comprising 27 positive and 224 negative genetic outcomes.

### Evaluation of LLMs’ Performance in Multidimensional Classification

V3.2-B demonstrated high accuracy (>90%) and *F*_1_-score (>0.9) across 4 fact-based classifications (Table 1). Specifically, its accuracy was 98.4% (250/254) for standardized terminology, 92.9% (236/254) for primary classification, 90.1% (229/254) for anatomical system, and 98.4% (250/254) for abnormality count. When independently applied to the entire dataset for these 4 objective tasks, V3.2-R achieved higher performance metrics across all categories compared to V3.2-B. However, because the performance of the base model was already exceptionally high, the improvements provided by the reasoning model were marginal (a maximum accuracy difference of 4.7% in the primary classification).

**Table 1.** Performance of the DeepSeek-V3.2 in classifying fetal ultrasound abnormalities (N=254).

Classification scheme	Accuracy (V3.2-B)^a^, n (%)	Accuracy (V3.2-R)^b^, n (%)	Precision	Recall	*F*_1_-score
Standardized terminology	250 (98.4)	254 (100)	0.99	0.99	0.99
Primary classification	236 (92.9)	248 (97.6)	0.94	0.96	0.95
Anatomical system	229 (90.1)	240 (94.4)	0.90	0.93	0.91
Abnormality count	250 (98.4)	254 (100)	0.99	0.96	0.98
Severity classification^c^	144 (56.6)	215 (84.6)	0.83^c^	0.77^c^	0.75^c^
Lethal (n=10)	—^d^	—	1.00	0.40	0.57
Major (n=31)	—	—	0.53	1.00	0.70
Minor (n=55)	—	—	0.79	0.82	0.80
Other (n=158)	—	—	1.00	0.85	0.92

a V3.2-B: DeepSeek-V3.2 base model.

b V3.2-R: DeepSeek-V3.2 reasoning-enhanced model.

c For the 4 factual classifications (excluding severity classification), the precision, recall, and *F*_1_-score metrics reported in the subsequent analysis are derived from V3.2-B, as its initial performance achieved the predefined high-level threshold of this study (*F*_1_-score >0.90). For the severity classification, the detailed performance metrics reported are based on V3.2-R, as the initial accuracy of V3.2-B was inadequate.

d Not applicable.

In contrast, the “Severity” classification proved more challenging. V3.2-B’s accuracy was only 56.7% (144/254), while V3.2-R significantly improved to 84.6% (215/254), with a macro-*F*_1_-score of 0.75.

A detailed breakdown of V3.2-R’s performance on the severity task revealed trade-offs: it achieved perfect precision for “lethal malformation” but with low recall (0.40), while for “major malformation,” it achieved perfect recall at the cost of lower precision (0.53). The LLM performed best when classifying findings into the “Other” category (*F*_1_-score=0.92).

### Comparative Efficacy of RAG Versus CoT Reasoning in Severity Assessment

We evaluated the performance of the V3.2-B and V3.2-R models across both the internal retrieval set and the external test set, before and after the implementation of RAG (Table 2).

**Table 2.** Comparative performance of DeepSeek-V3.2 base and reasoning models for fetal anomaly severity assessment before and after RAG^a^.

Model and set	Before RAG^b^ (n=127)				After RAG (n=127)				*P* value^c^
	Accuracy	Precision	Recall	*F*_1_-score	Accuracy	Precision	Recall	*F*_1_-score	
V3.2-B^d^									
Retrieval	0.56	0.45	0.62	0.48	0.70	0.61	0.68	0.61	.002
Lethal (n=4)	—^e^	0.57	1.00	0.72	—	0.50	0.75	0.60	—
Major (n=19)	—	0.29	0.84	0.43	—	0.40	0.79	0.54	—
Minor (n=26)	—	0	0	0	—	0.62	0.38	0.48	—
Other (n=78)	—	0.96	0.65	0.78	—	0.90	0.78	0.84	—
Test	0.57	0.43	0.62	0.46	0.59	0.49	0.59	0.49	.61
Lethal (n=6)	—	0.54	1.00	0.71	—	0.44	0.67	0.53	—
Major (n=12)	—	0.18	0.75	0.29	—	0.25	0.75	0.35	—
Minor (n=29)	—	0	0	0	—	0.30	0.24	0.27	—
Other (n=80)	—	0.98	0.71	0.83	—	1.00	0.69	0.81	—
V3.2-R^f^									
Retrieval	0.83	0.83	0.72	0.70	0.99	0.99	1.00	0.99	<.001
Lethal (n=4)	—	1.00	0.25	0.40	—	1.00	1.00	1.00	—
Major (n=19)	—	0.58	1.00	0.73	—	1.00	1.00	1.00	—
Minor (n=26)	—	0.75	0.81	0.78	—	0.96	1.00	0.98	—
Other (n=78)	—	1.00	0.83	0.91	—	1.00	0.99	0.99	—
Test	0.86	0.83	0.80	0.77	0.81	0.69	0.69	0.69	.33
Lethal (n=6)	—	1.00	0.50	0.67	—	0.60	0.50	0.50	—
Major (n=12)	—	0.48	1.00	0.65	—	0.53	0.67	0.59	—
Minor (n=29)	—	0.83	0.83	0.83	—	0.71	0.69	0.70	—
Other (n=80)	—	1.00	0.87	0.93	—	0.90	0.90	0.90	—

a Data represent the performance metrics for the internal retrieval set (n=127) and the external test set (n=127). The retrieval set consists of data used to construct the RAG vector database, whereas the test set comprises the unseen data.

b RAG: retrieval-augmented generation.

c *P* values were calculated using the McNemar test to determine the statistical significance of the difference in overall accuracy before and after the implementation of RAG for each model.

d V3.2-B: DeepSeek-V3.2 base model.

e Not applicable.

f V3.2-R: DeepSeek-V3.2 reasoning-enhanced model.

V3.2-R demonstrated a substantial performance advantage over V3.2-B prior to the application of RAG. On the test set, V3.2-R achieved an accuracy of 86%, significantly outperforming V3.2-B, which achieved only 57%. Notably, in the classification of “Minor” anomalies, V3.2-B completely failed to identify any cases (precision, recall, and *F*_1_-score=0), whereas V3.2-R achieved a high *F*_1_-score of 0.83. This disparity highlights the intrinsic limitation of V3.2-B in handling subjective severity grading without explicit reasoning capabilities.

When applied to the retrieval set, RAG significantly improved the performance of both models. The accuracy of V3.2-B increased from 56% to 70% (*P*=.002), with the *F*_1_-score for “Minor” anomalies rising from 0 to 0.48, indicating that the model successfully retrieved relevant examples to correct its output. Moreover, V3.2-R achieved near-perfect performance with RAG, improving accuracy from 83% to 99% (*P*<.001). Precision and recall metrics across all severity subtypes approached or reached 1.00. These results confirm that the RAG pipeline was technically functional and capable of enhancing performance when the test data were semantically identical or highly similar to the knowledge base.

Crucially, the performance gains observed in the retrieval set did not translate to the external test set, revealing a critical limitation in the generalizability of RAG for this specific task. V3.2-B showed no statistically significant improvement with RAG (accuracy: 57% vs 59%, *P*=.61). While RAG slightly improved the detection of “Minor” anomalies (*F*_1_-score increased to 0.27), the overall capability remained suboptimal compared to the reasoning model. The implementation of RAG on V3.2-R resulted in a slight, though not statistically significant, decline in accuracy on the external test set (86% vs 81%, *P*=.33). Specifically, the *F*_1_-scores for “Lethal” and “Major” anomalies decreased after RAG (lethal: 0.67-0.50; major: 0.65-0.59).

These data indicate that while RAG can effectively guide LLMs to memorize or retrieve specific patterns within the knowledge base, it fails to significantly enhance—and may potentially hinder—performance on unseen, heterogeneous clinical data. In contrast, the CoT reasoning inherent in V3.2-R (without RAG) proved to be the most robust approach for the subjective task of severity assessment, achieving the highest stand-alone accuracy (86%) and *F*_1_-scores on the external validation cohort.

### Descriptive Statistics of Prenatal Ultrasound Abnormalities

**Figure 2.**
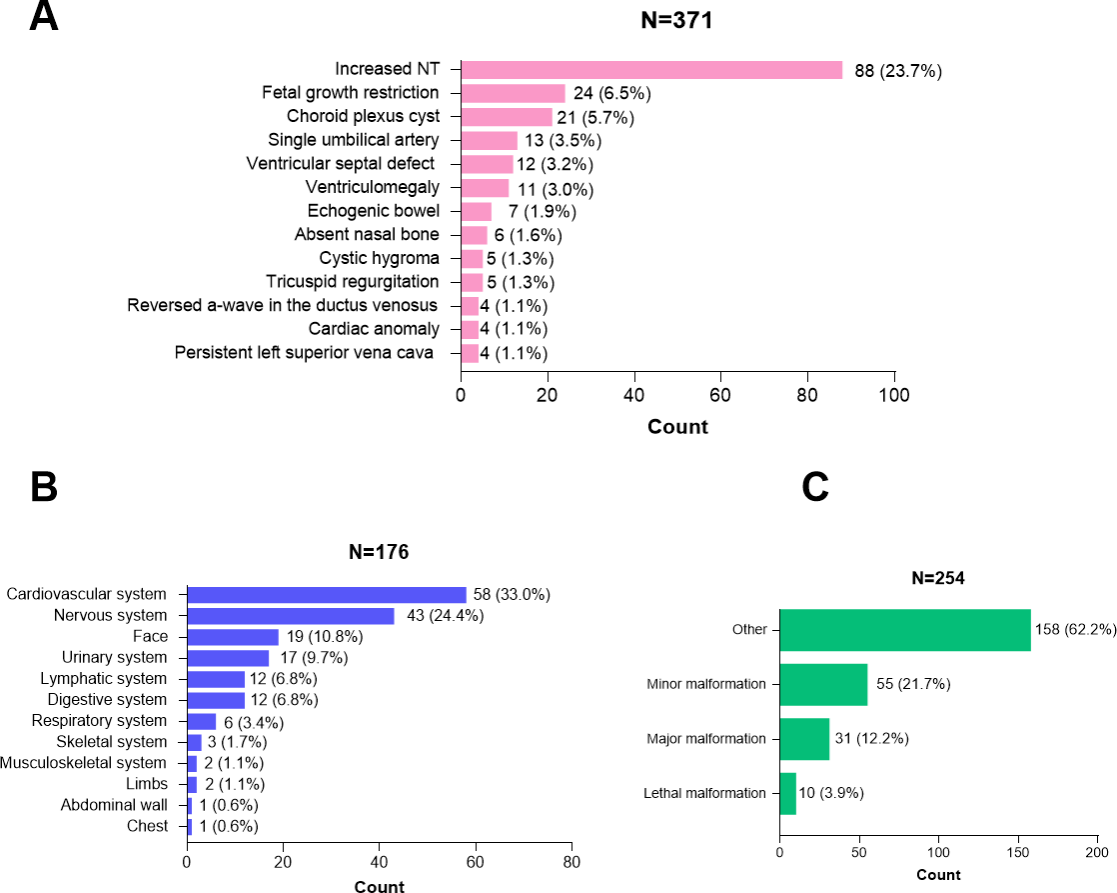
Distribution of prenatal ultrasound abnormalities based on the manually curated classifications generated by DeepSeek-V3.2. (A) Frequencies of standardized medical terms for ultrasound findings. The model standardized unstructured descriptions into common terms, generating 371 entries in total. Only terms with a frequency greater than 1% are displayed. (B) Distribution of abnormalities by the affected anatomical system (n=176). (C) Distribution of cases by severity classification (n=254).

A descriptive analysis of the curated dataset revealed the distribution of abnormalities across the 5 classification schemes (Figure 2).

By standardized terminology, the most frequent among the 371 identified findings were increased NT (88/371, 23.7%), FGR (24/371, 6.5%), choroid plexus cyst (21/371, 5.7%), and single umbilical artery (13/371, 3.5%; Figure 2A). Most reports described solitary findings (171/254, 67.3%) rather than multiple findings (83/254, 32.7%). Among 176 classified structural anomalies, the cardiovascular (58/176, 33%) and nervous (43/176, 24.4%) systems were the most commonly affected (Figure 2B). The primary classifications were distributed among increased NT (96/292, 32.5%), other soft markers (83/292, 28.4%), structural abnormalities (82/292, 28.1%), and FGR (32/292, 11%). Finally, by severity, most cases were categorized as “Other” (158/254, 62.2%), followed by minor (55/254, 21.7%), major (31/254, 12.2%), and lethal (10/254, 3.9%) malformations (Figure 2C).

### Association Analysis

**Figure 3.**
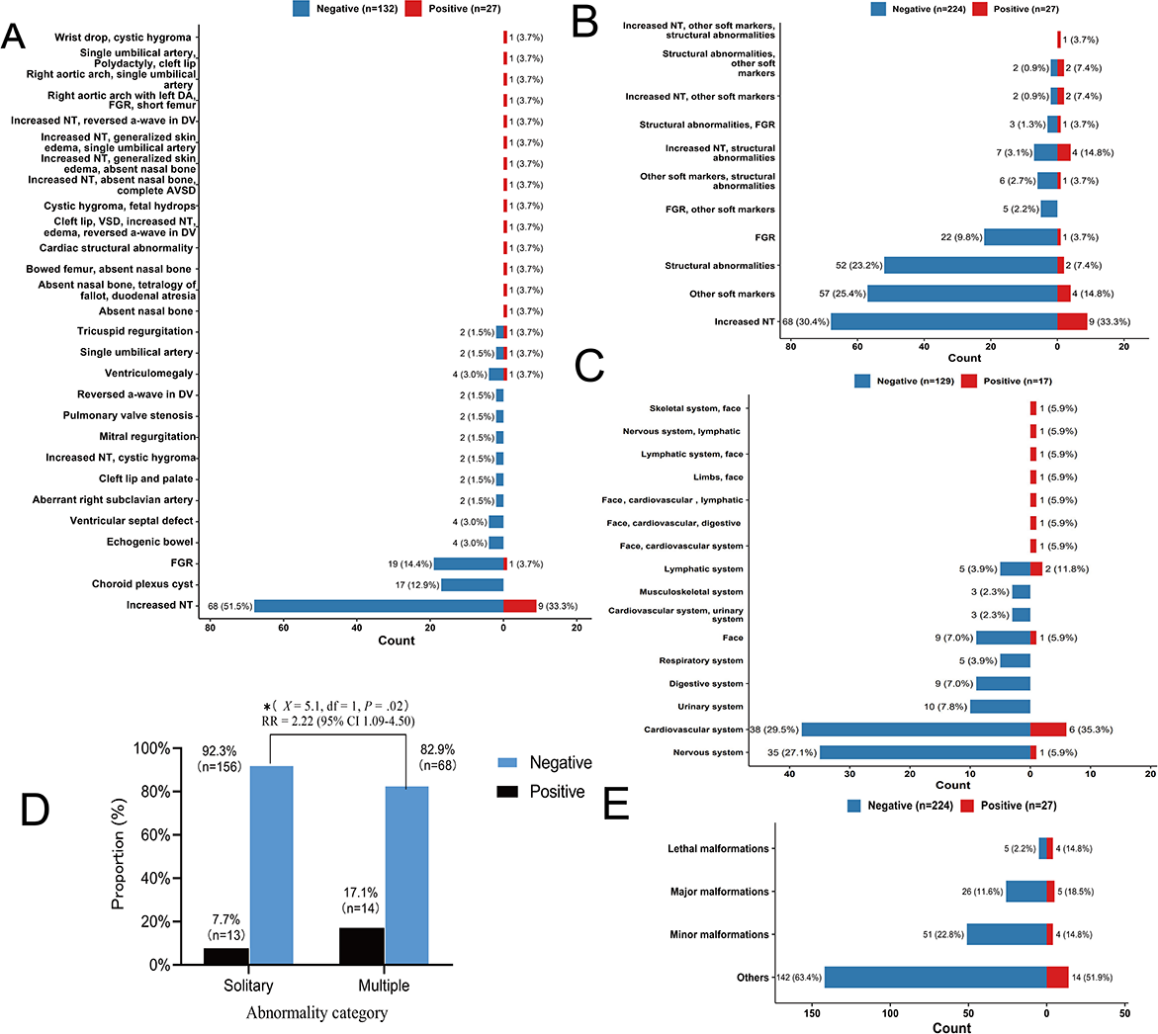
Association analysis between classified prenatal ultrasound abnormalities and genetic diagnostic outcomes. The bar charts display the number and proportion of cases with negative (blue) and positive (red) prenatal diagnoses for each classification scheme. (A) Analysis by standardized terminology. This panel shows all 27 positive cases and their associated ultrasound findings. For clarity, only findings from the negative diagnosis group with a frequency greater than 2 are displayed. (B) Analysis by primary classification. (C) Analysis by the anatomical system. (D) Analysis by the abnormality count. The rate of positive diagnoses was significantly higher in cases with multiple abnormalities compared to those with solitary findings (*P*=.02, Pearson *χ*^2^ test). (E) Analysis by severity (subjective assessment). AVSD: atrioventricular septal defect; DA: ductus arteriosus; DV: ductus venosus; FGR: fetal growth restriction; NT: nuchal translucency; RR: relative risk; VSD: ventricular septal defect.

An association analysis correlated the classified ultrasound findings with genetic outcomes (Figure 3). The presence of multiple abnormalities significantly increased the risk of a positive genetic diagnosis (Figure 3) compared to solitary findings (14/82, 17.1% vs 13/169, 7.7%).

Among solitary findings, the increased NT was the most common abnormality associated with a positive genetic diagnosis (n=9; Figures 3A and 3B). Notably, no positive diagnoses were found in cases with isolated choroid plexus cysts, ventriculomegaly, echogenic bowel, or ventricular septal defects (Figure 3A).

Risk is also correlated with the anatomical system. Among cases with positive genetic diagnoses, cardiovascular and lymphatic system abnormalities were the most frequent. Conversely, no positive diagnoses in this cohort were associated with isolated anomalies of the urinary, digestive, or musculoskeletal systems (Figure 3C).

More importantly, lethal and major malformations were disproportionately represented in the positive diagnosis cohort (Figure 3E), accounting for 14.8% (4/27) and 18.5% (5/27) of positive cases, respectively. In addition, positive cases constituted 44.44% (4/9) of all lethal malformations and 16.13% (5/31) of all major malformations but only 7.14% (4/56) of minor malformations.

## Discussion

### Principal Results

This study establishes an adaptive “fast-slow” framework utilizing the open-source DeepSeek-V3.2 family for the automated, multidimensional classification of prenatal ultrasound reports. By strategically deploying a high-speed base model for factual extraction and a reasoning-enhanced model for subjective assessment, our approach significantly enhances data annotation efficiency while resolving the complexity of phenotype validation. Crucially, we identified a pivotal mechanistic dichotomy; while RAG improves performance on the data seen within the knowledge base, it fails to generalize to external subjective tasks. In contrast, CoT reasoning demonstrates superior robustness in “unseen” scenarios, effectively mimicking the “System 2” clinical judgment required for severity grading. This work provides a foundational pipeline for phenotype-driven research using unstructured hospital data and offers a reliable tool to support clinical decision-making, highlighting the necessity of matching model cognitive architectures to clinical task complexity.

### Limitations

This study has limitations. First, while we highlighted the superiority of CoT over RAG for subjective tasks, our RAG implementation utilized a specific reranking strategy (Qwen3-Reranker). Alternative retrieval algorithms or hybrid approaches might yield different results. Second, the cohort, while expertly annotated, is relatively small (n=254) and derived from a single center, and the fact that the “gold standard” annotations were established through the expert review of model outputs rather than being fully independent inevitably introduces a degree of subjectivity. Consequently, certain clinically important subcategories, such as lethal malformations, are represented by very small sample sizes, which may affect the statistical stability of our performance metrics within these subgroups. Furthermore, our evaluation utilized an internal sequential split. While the strict separation of patient IDs prevented direct data leakage, the unseen test set shared local linguistic patterns with the retrieval knowledge base. This setup inherently provides an optimistic performance estimate for the RAG pipeline. However, this constraint actually reinforces our primary findings; even under conditions highly favorable to retrieval, CoT reasoning still demonstrated superior robustness for subjective tasks. While this single-center design accurately simulates local clinical deployment—where a hospital utilizes its own historical records—it does not evaluate true out-of-distribution generalizability. Broader generalizability across different institutional reporting styles remains to be established through future multicenter or larger-scale studies. Third, we did not formally evaluate prompt engineering variations [21-23], although reasoning models typically show resilience to prompt nuances [24]. Fourth, potential confounders, such as gestational age, were not integrated into the model’s decision logic, warranting future multimodal investigations. Finally, it is also important to acknowledge limitations regarding our clinical end points. For one, treating all low-risk NIPT2.0 cases as negative genetic outcomes without universal confirmatory amniocentesis—while clinically justified by the test’s high negative predictive value and our cohort observations—may introduce a minor risk of misclassification bias. Statistically, this inherent uncertainty of screening-based negatives could potentially lead to a slight underestimation of the true genetic risk associated with specific ultrasound phenotypes. Furthermore, our validation specifically targeted pathogenic genetic risk to support invasive diagnostic decision-making. Because ultrasound anomalies frequently arise from heterogeneous nongenetic etiologies, a more comprehensive assessment of the framework’s overall clinical utility will require future studies integrating additional longitudinal end points, such as postnatal diagnoses and long-term functional outcomes.

### Comparison With Prior Work

While LLMs have shown broad capabilities across medicine [25-47], a “one-size-fits-all” approach remains inefficient for complex clinical workflows. Our findings challenge the prevailing assumption that RAG is the universal solution for medical LLM hallucinations. In our study, RAG successfully corrected the base model’s errors within the retrieval set, confirming its utility for pattern matching. However, this performance collapsed on the external test set. This suggests that for subjective tasks like severity assessment—which rely on synthesizing subtle cues—semantic retrieval is insufficient. RAG retrieves similar text chunks but not necessarily the logic of the diagnosis. Conversely, the V3.2-R model, utilizing CoT, achieved an 86% accuracy on the external set without accessing the knowledge base. This indicates that internalized reasoning capabilities (navigating clinical logic steps) are more critical than external knowledge retrieval (accessing facts) when dealing with the nuanced subjectivity of fetal anomalies [44]. Notably, introducing RAG to V3.2-R degraded performance to 81%, suggesting potential noise interference.

Unlike commercial proprietary models, the open-source nature of the DeepSeek suite allows for secure local deployment, ensuring patient data privacy—a nonnegotiable requirement for handling sensitive prenatal records [39,40,48-51]. Our framework maximizes resource efficiency: by routing the majority of straightforward tasks (entity extraction and counting) to the “Fast” V3.2-B model, we preserve the computationally expensive “Slow” V3.2-R model only for tasks where it provides a statistically significant benefit. This tiered approach addresses the processing speed bottlenecks often cited as a barrier to deploying reasoning models in real-time clinical settings [52,53]. Admittedly, the DeepSeek LLMs in this study did not achieve perfect accuracy across all classification tasks; even V-3.2R achieved only 84.6% accuracy in the subjective severity grading of the entire dataset. However, model performance is expected to improve with future advancements in open-source LLMs. Furthermore, while no current LLM can fully replace specific clinical practice, they are already sufficient to significantly enhance efficiency.

The ultimate value of this technical framework lies in its clinical utility. By enabling high-throughput, multidimensional classification, we were able to conduct an association analysis between sonographic phenotypes and genetic outcomes. However, these association results are exploratory and specific to our single-center cohort. Establishing higher-level evidence for the strength of phenotype-genotype correlations would necessitate much larger, multicenter datasets and more rigorous statistical validation to ensure broader clinical applicability. The fundamental aim of this analysis was to illustrate the practical clinical significance of the multidimensional profiles generated by our LLM framework. Our automated severity grading successfully stratified patients into distinct risk categories, confirming established high-risk predictors, such as multiple anomalies and specific system involvement (eg, cardiovascular) [6,54-58]. The decreasing genetic risk observed across lethal (4/9, 44.44%), major (5/31, 16.13%), and minor (4/56, 7.14%) malformations demonstrates that subjective severity grading is an indispensable dimension for phenotype-driven diagnosis. Importantly, the reasoning model accurately identified “Minor” malformations—a category the base model completely missed. This granularity provides quantitative support for the lower (yet nonnegligible) risk nature of isolated markers, potentially aiding in reducing unnecessary invasive procedures for low-risk findings [59], while ensuring that subtle but significant patterns are not overlooked [60]. This validates that our “human-in-the-loop” LLM framework does not merely digitize text but actively contributes to refining risk stratification.

### Conclusions

This study demonstrates that a monolithic LLM strategy is insufficient for the diverse challenges of prenatal diagnosis. We propose an adaptive framework where “Fast” models handle factual extraction, while “Slow” reasoning models are prioritized for subjective clinical assessment, as they demonstrated greater robustness than our specific RAG implementation in this cohort. However, this finding is contextualized within our current experimental framework and does not constitute a generalized conclusion regarding the inherent superiority of CoT over RAG. By aligning the cognitive architecture of LLM agents with the cognitive demands of medical tasks, we offer a scalable, privacy-preserving path to transform unstructured ultrasound narratives into actionable, phenotype-driven clinical intelligence.

## Supplementary material

10.2196/91399 Multimedia Appendix 1 The full anonymized prenatal-ultrasound abnormality dataset, 5 classification schemes with expert scores, DeepSeek large language model prompts, and severity assessment before and after RAG.

## Abbreviations

CoT: chain-of-thought
FGR: fetal growth restriction
LLMs: large language models
NIPT2.0: enhanced noninvasive prenatal test
NT: nuchal translucency
RAG: retrieval-augmented generation
